# Alpha-1 Adrenergic Receptors Modulate Glutamate and GABA Neurotransmission onto Ventral Tegmental Dopamine Neurons during Cocaine Sensitization

**DOI:** 10.3390/ijms21030790

**Published:** 2020-01-25

**Authors:** Maria Carolina Velasquez-Martinez, Bermary Santos-Vera, Maria E. Velez-Hernandez, Rafael Vazquez-Torres, Carlos A. Jimenez-Rivera

**Affiliations:** 1 Grupo de Neurociencias y Comportamiento, Departamento de Ciencias Básicas, Facultad de Salud, Universidad Industrial de Santander, Bucaramanga 680006, Colombia; macarvel@uis.edu.co; 2 Department of Biology, Cayey Campus, University of Puerto Rico, Cayey, PR 00737, USA; bermary.santos@upr.edu; 3 Department of Biological and Health Sciences, Texas A&M University-Kingsville, Kingsville, TX 78363, USA; Maria.Hernandez-Velez@tamuk.edu; 4 Department of Physiology, Medical Sciences Campus, University of Puerto Rico, San Juan, PR 00925, USA; rvazquez20@hotmail.com

**Keywords:** alpha1 adrenergic receptors, GABA, glutamate, cocaine sensitization, ventral tegmental area, dopamine neurons, noradrenergic inputs

## Abstract

The ventral tegmental area (VTA) plays an important role in the reward and motivational processes that facilitate the development of drug addiction. Presynaptic α1-AR activation modulates glutamate and Gamma-aminobutyric acid (GABA) release. This work elucidates the role of VTA presynaptic α1-ARs and their modulation on glutamatergic and GABAergic neurotransmission during cocaine sensitization. Excitatory and inhibitory currents (EPSCs and IPSCs) measured by a whole cell voltage clamp show that α1-ARs activation increases EPSCs amplitude after 1 day of cocaine treatment but not after 5 days of cocaine injections. The absence of a pharmacological response to an α1-ARs agonist highlights the desensitization of the receptor after repeated cocaine administration. The desensitization of α1-ARs persists after a 7-day withdrawal period. In contrast, the modulation of α1-ARs on GABA neurotransmission, shown by decreases in IPSCs’ amplitude, is not affected by acute or chronic cocaine injections. Taken together, these data suggest that α1-ARs may enhance DA neuronal excitability after repeated cocaine administration through the reduction of GABA inhibition onto VTA dopamine (DA) neurons even in the absence of α1-ARs’ function on glutamate release and protein kinase C (PKC) activation. α1-AR modulatory changes in cocaine sensitization increase our knowledge of the role of the noradrenergic system in cocaine addiction and may provide possible avenues for therapeutics.

## 1. Introduction

The mesocorticolimbic system is composed of dopamine (DA) neurons projecting mainly from the ventral tegmental area (VTA) to cortical and ventral forebrain structures [1,2,3]. The activation of VTA DA neurons has been implicated in motivated behaviors as well as in mediating the reinforcing actions of drug abuse [4,5,6]. VTA DA neurons receive noradrenergic (NE) inputs from the locus coeruleus and other pontine structures [7,8], and tracing studies have shown that NE afferents have extrasynaptic and synaptic connections on VTA DA neurons [9]. Moreover, the VTA contains alpha-1 adrenoreceptors (α1-ARs) [10], which are located primarily in presynaptic elements [11,12,13]. NE inputs have been shown to facilitate VTA DA neuronal transmission and induce changes in burst firing via α1-ARs [14,15,16]. Also, α1-ARs participate in the development of stress and anxiety responses, and in addiction-related behaviors as well [17,18,19,20]. Furthermore, activation of α1-ARs at presynaptic terminals increase glutamate (Glu) and decreased GABA release onto VTA DA neurons, changing their excitability [12,13].

Diverse brain nuclei send Glu and GABAergic inputs to the VTA. The Glu innervation to the VTA includes afferents from the prefrontal cortex, the lateral hypothalamus, medial habenula, bed nucleus of the stria terminalis, dorsal raphe, laterodorsal, and pedunculopontine tegmental nuclei [21,22,23,24,25,26,27]. Also, the presence of local Glu neurons has been demonstrated [28,29]. On the other hand, the mesopontine tegmentum, the lateral habenula via the rostromedial tegmentum, nucleus accumbens, and the periaqueductal gray provide GABAergic innervation to the VTA [30,31,32].

Chronic cocaine exposure leads to a progressive increase in locomotor activity in rats termed, behavioral sensitization [33,34]. As a result of chronic drug exposure, neuroadaptations in mesocorticolimbic synapses, including DA, Glu, and GABA neurotransmission, occur [35,36,37]. In the VTA, DA cell activation plays an important role in cocaine sensitization [38,39]. For example, increases in the AMPA/NMDA ratio [39,40,41] and VTA DA firing [42] develop during cocaine sensitization. On the other hand, daily exposure to cocaine reduces the GABA_A_ receptor-mediated inhibition of DA neurons facilitating neuronal excitation [43]. Cocaine-induced inhibition of monoamine reuptake may further enhance the availability of noradrenaline at synapses and cause stronger stimulation of α1-ARs [44]. Also, postsynaptic α1-ARs activation on DA neurons contributes to increase DA levels, motor activity, and enhance the pacemaker and burst firing frequency of VTA cells [45,46]. α1b-AR knockout animals and α1-AR blockade demonstrate the critical role of this receptor for the development of addictive processes related to cocaine sensitization and self-administration [19,47,48,49]. Furthermore, prazosin (α1-AR antagonist) attenuates a cocaine-induced reinstatement of drug-seeking behavior in rats [46,50]. Prazosin can also reduce relapse in cocaine addicts [51]. Moreover, α1-ARs’ density changes in different rat brain regions after cocaine sensitization [52]. Investigations have shown that decreased α1-AR regulation of an mGlu-R-mediated current might be a cellular mechanism associated with the effects of cocaine withdrawal in a self-administration protocol [53]. In sum, these studies suggest that α1-ARs contribute to the behavioral and neurochemical effects of cocaine addiction.

Electrophysiological studies of VTA α1-AR direct modulation on Glu and GABA terminals at different stages of cocaine sensitization have not been reported. Here, we tested the contribution of presynaptic α1-ARs activation in VTA DA neuronal excitability: glutamatergic and GABAergic modulation after acute and chronic cocaine treatment and after a withdrawal period.

## 2. Results

### 2.1. Presynaptic α1-AR Modulation of Glutamate Release on VTA DA Neurons after Acute Cocaine Injection

**Figure 1:**
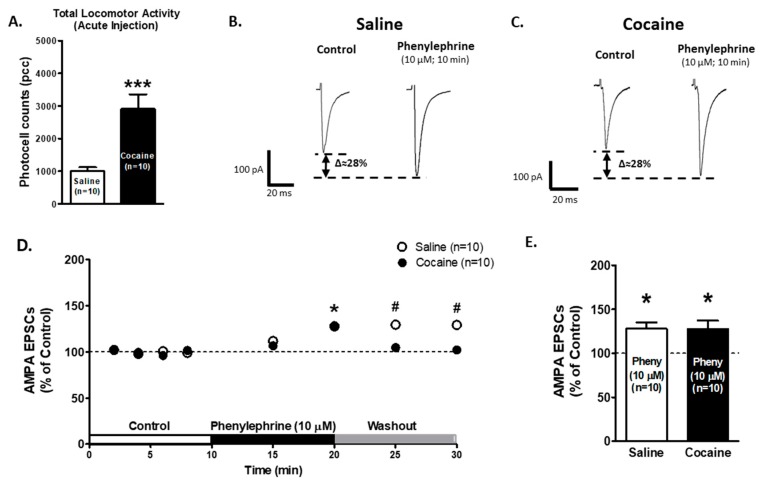
α1-ARs-mediated effect on AMPA EPSCs is preserved in ventral tegmental area (VTA) dopamine (DA) neurons after one cocaine/saline injection. (**A**) The bar graph shows total locomotor activity recorded from saline- (0.9%, white bar; *n* = 10) and cocaine-treated (15 mg/kg i.p., black bar; *n* = 10) animals. The locomotion of cocaine-treated animals was significantly increased when compared to saline-treated controls (Unpaired *t*-test *p* < 0.001). (**B**,**C**) Representative recordings from saline- and cocaine-treated animals, respectively, showing that phenylephrine superfusion (10 μM, 10 min) induces a significant increase in AMPA EPSCs amplitude in VTA DA neurons in both groups. (**D**) A time course summary of phenylephrine’s effect on AMPA EPSCs recorded from VTA DA neurons from saline- (*n* = 10) and cocaine- (*n* = 10) treated animals at 8 min from control recordings (2 min intervals), 5 and 10 min phenylephrine (10 μM), and 5 and 10 min washout (One-way ANOVA, Newman-Keuls post-hoc). (**E**) The bar graph shows that 10 min of phenylephrine application resulted in a ~28% increase in AMPA EPSCs amplitude in VTA DA neurons from saline- (*n* = 10/10) and cocaine- (*n* = 10/10) treated animals when compared to control recordings (One-way ANOVA, Newman-Keuls post-hoc.). One-way ANOVA, Newman-Keuls post-hoc * *p* < 0.05. Unpaired *t*-test *** *p* < 0.001. Two-way repeated-measures ANOVA, Bonferroni post-hoc # *p* < 0.05. *n* = the number of cells recorded/number of animals.

We first tested the effect of α1-ARs activation on glutamate release in animals treated with one cocaine injection. Animals were injected with either saline (0.9%; *n* = 10) or cocaine (15 mg/kg, i.p.; *n* = 10), and motor activity was recorded at 10 min intervals for 60 min. For the total activity (Figure 1A), an unpaired *t*-test (df = 18, *t* = 4.104, *p* = 0.0007) showed differences between saline (*n* = 10; 1003 ± 118.6 photocell counts) and cocaine (*n* = 10; 2907 ± 448.4 photocell counts) treated animals. These results indicate that acute cocaine-treated animals present the characteristic of an increased locomotion response to cocaine. 

Twenty-four hours after the injection, brain slices were prepared and AMPA EPSCs were recorded on VTA DA neurons from saline- and cocaine-treated animals. Ten minutes of phenylephrine superfusion (10 μM) increased AMPA EPSCs amplitude in both animal groups: saline group: 127.76 ± 7.22% of control (*n* = 10; One-way ANOVA F2,29 = 4.81, *p* < 0.05, Figure 1B,C); cocaine group: 127.99 ± 9.13% of control (*n* = 10; One-way ANOVA F2,29 = 7.14, *p* < 0.05, Figure 1C–E). No differences were found between the DA-cells of saline- and cocaine-treated animals after 10 min of phenylephrine superfusion (Two-way repeated-measures ANOVA, treatment factor F1,126 = 2.092, *p* = 0.16, time factor F7,126 = 8.922, *p* < 0.0001, interaction F7, 17 = 2.96. *p* < 0.01). Although, Bonferroni post hoc test detected differences between treatments (saline vs cocaine) after 5 and 10 min of washout (*p* < 0.05 and *p* < 0.01, respectively). These results suggest that α1-ARs are functional after one cocaine injection.

### 2.2. α1-ARs-Mediated AMPA EPSCs Effect is Absent in VTA DA Neurons from Cocaine Sensitized Animals

**Figure 2:**
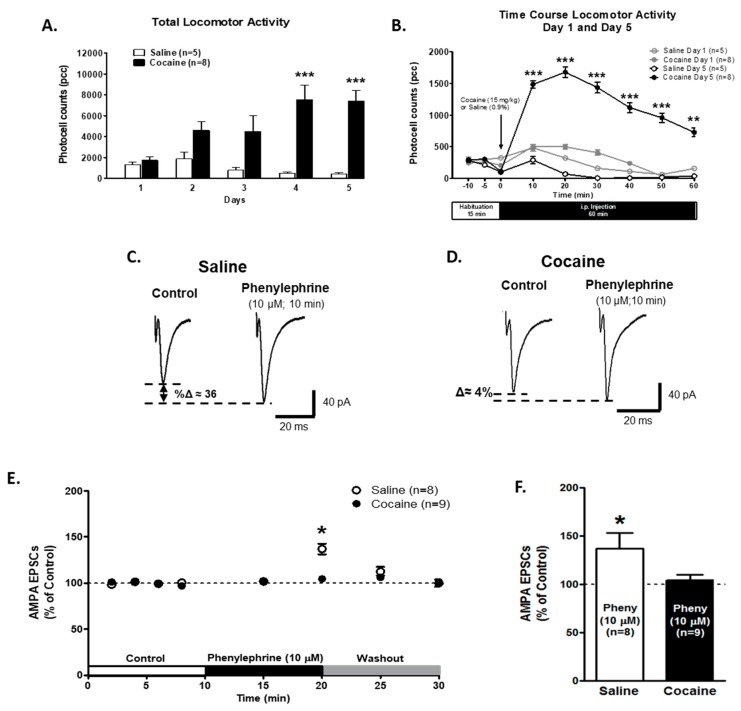
α1-ARs-mediated effect on AMPA EPSCs is absent in the VTA DA neurons of cocaine sensitized animals. (**A**) The bar graph shows the total locomotor activity recorded from saline- (0.9%, white bars; *n* = 5) and cocaine- (15 mg/kg i.p., black bars; *n* = 8) treated animals. Cocaine-injected animals show a progressive increase in locomotor activity over 5 days. On days 4 and 5, the total activity was significantly increased when compared to day 1 in cocaine-treated animals (Two-way repeated measures ANOVA, Bonferroni post hoc test *p* < 0.001). (**B**) The time course of locomotor activity on days 1 (grey) and 5 (black) of cocaine- (15 mg/kg, i.p.) (filled) or saline- (unfilled) treated animals. Cocaine sensitization was manifested as an increase in locomotor activity during the first 10–50 min after cocaine injection in comparison with day 1. The data (pcc/60 min; Mean ± SEM) were analyzed by Two-way repeated measures ANOVA, Bonferroni post hoc test (*p* < 0.001). (**C**,**D**) Representative recordings from saline- and cocaine-treated animals, respectively, illustrating that phenylephrine superfusion (10 μM, 10 min) induces a significant increment in AMPA EPSCs amplitude only in VTA DA neurons from saline-treated animals. (**E**) A time course summary of the effects of phenylephrine bath application on AMPA EPSCs amplitude recorded from VTA DA neurons from saline-treated animals (*n* = 8/5) and VTA DA neurons from cocaine-treated animals (*n* = 9/8) at 8 min after control recording (2 min intervals), 5 and 10 min phenylephrine (10 μM), and 5 and 10 min washout. A 10 min phenylephrine application increased the AMPA EPSCs amplitude only in neurons from saline-treated animals compared to control recordings (One-way ANOVA, Newman-Keuls post-hoc, *p* < 0.05). A washout of phenylephrine’s response was observed. (**F**). A bar graph showing that, in neurons from saline-treated animals (*n* = 8), phenylephrine application resulted in an ~36% increase in AMPA EPSCs amplitude compared to control recordings (One-way ANOVA, Newman-Keuls post-hoc, *p* < 0.05). No significant differences were observed on VTA DA neurons in cocaine-treated animals (*n* = 9) after 10 min of phenylephrine application (104.6 ± 5.3%). Two-way repeated measures ANOVA showed in factor time, at 10 min of phenylephrine superfusion (F7,105 = 2.831, Bonferroni post hoc, *p* < 0.05) * *p* < 0.05; ** *p* < 0.01, *** *p* < 0.001. *n* = the number of cells recorded/number of animals.

To determine if presynaptic α1-AR modulation on glutamate release was altered in VTA DA neurons after behavioral sensitization, animals were injected with either saline (0.9%; *n* = 5) or cocaine (15 mg/kg, i.p.; *n* = 8) for five consecutive days. For total activity (Figure 2A), Two-way repeated measures ANOVA showed differences in treatment: saline vs. cocaine (F1,44 = 14.80; *p* = 0.0027). Also, total activity between the days (F4,44 = 4.09; *p* = 0.0066) and the interaction between treatment and days showed differences (F4,44 = 7.41; *p* = 0.0001). A Bonferroni post hoc analysis revealed that cocaine-treated animals had a higher total activity than saline-treated animals on days 4 (*p* = 0.001) and 5 (*p* = 0.001). The time course of locomotor responses (Figure 2B) compares day 1 and day 5 after saline or cocaine injection. Two-way repeated measures ANOVA showed differences in treatment (F3,110 = 23.22; *p* < 0.0001) and time (F5,110 = 13.661; *p* < 0.0001). The interaction between time and treatment showed significant differences (F15,110 = 2.21, *p* < 0.01). The Bonferroni post hoc analysis demonstrated an increase in locomotor activity on day 5 of cocaine treatment during the 60 min recording after cocaine injection compared to one day of cocaine treatment (*p* < 0.001). Together, these results indicate that the animals used in this study were successfully sensitized to cocaine.

Recordings of VTA DA neurons from saline-treated animals show that a bath application of the selective α1-AR agonist, phenylephrine (10 μM), for 10 min, but not for 5 min, increased AMPA EPSCs peak amplitude to 136.9 ± 16.2% of control (*n* = 8; Figure 2C–F). However, on VTA DA neurons from 5-days cocaine-treated animals, phenylephrine superfusion was unable to exert its excitatory action on AMPA EPSCs after a 5- or 10-min application (control 99.9 ± 1.04%; phenylephrine 10 min 104.6 ± 5.33%; *n* = 9; Two-way repeated measures ANOVA treatment factor F1.105 = 0.644, *p* = 0.43; time factor F7,105 = 2.831, *p* < 0.01; interaction F7,105 = 1.64, *p* = 0.13; Bonferroni post hoc, *p* < 0.05, Figure 2E,F). These results strongly suggest that α1-ARs have been altered by 5 days of cocaine administration.

### 2.3. Presynaptic PKC Activity is Decreased in VTA Slices of Cocaine Sensitized Animals

**Figure 3:**
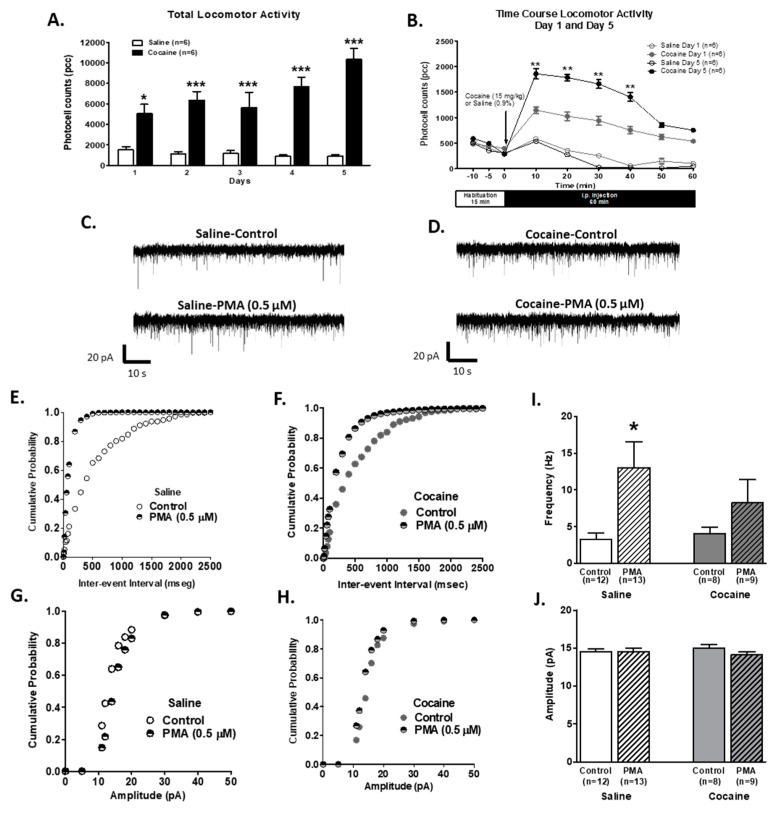
Phorbol 12-myristate 13-acetate (PMA) pre-treatment on VTA DA neurons from cocaine-sensitized animals failed to increase sEPSCs frequency or amplitude. (**A**) The bar graph shows the total locomotor activity recorded from saline- (0.9%, white bars; *n* = 6) and cocaine- (15 mg/kg i.p., black bars; *n* = 6) treated animals. Cocaine-injected animals showed a progressive increase in locomotor activity over days. On day 5, total activity was significantly increased when compared to day 1 in cocaine-treated animals (Two-way repeated measures ANOVA, time F4,40 = 3.69, *p* < 0.05; treatment F1,40 = 78.72, *p* < 0.001). (**B**) The time course of locomotor activity on days 1 (grey) and 5 (black) of cocaine- (15 mg/kg, i.p.; filled; *n* = 6) or saline- (unfilled; *n* = 6) treated animals. Cocaine sensitization was manifested as an increase in locomotor activity during the first 40 min after cocaine injection in comparison with day 1. The data (pcc/60 min; Mean ± SEM) were analyzed by Two-way repeated measures ANOVA, time F8, 160 = 33.99, treatment F3,160 = 25.97, *p* < 0.001, Bonferroni post hoc test, *p* < 0.01. (**C**). Representative recordings from the neurons of saline-treated animals, illustrating that PMA pretreatment (0.5 μM; 30 min) increases sEPSC frequency but not the amplitude. (**D**). Representative recordings from neurons of cocaine-sensitized animals illustrating that PMA pretreatment failed to increase the frequency or amplitude of sEPSCs. (**E**) and (**G**) The cumulative probability of the inter-event interval and amplitude cumulative distribution, respectively, from control- and PMA-treated neurons of saline-injected animals. Plots were constructed from the cells used in (**C**,**F**,**H**). The cumulative probability of inter-event interval and amplitude cumulative distribution (respectively) from control- and PMA-treated neurons of cocaine-sensitized animals. The plots were constructed from the cells used in (**D**,**I**). A summary graph showing that PMA treatment increased the mean sEPSC frequency in VTA DA cells from saline- (*n* = 13/6) but not cocaine- (*n* = 9/6) injected animals. (**J**) Summary graph shows that PMA treatment failed to increase the mean sEPSC amplitude in VTA DA cells from saline- (*n* = 8/6) and cocaine- (*n* = 9/6) injected animals. An unpaired *t*-test. * *p* < 0.05, ** p < 0.01, *** *p* < 0.001. *n* = the number of cells recorded/number of animals.

To determine the mechanism of α1-AR physiological alteration by the cocaine sensitization process, we recorded spontaneous EPSCs (sEPSCs) from VTA DA neurons of saline- and cocaine-treated animals in the presence of a PKC activator. For this, rats were injected with either saline (0.9%; *n* = 6) or cocaine (15 mg/kg, i.p.; *n* = 6) for five consecutive days. Animals treated with 5 days of cocaine showed an increase the in total locomotor activity in comparison to saline-injected animals (two-way repeated measures ANOVA, treatment F1,10 = 78.72, *p* < 0.0001; time F4,10 = 3.69, *p* < 0,05, Figure 3A). Bonferroni post hoc analysis revealed that cocaine treated animals had higher total activity on day 5 than day 1 (*p* < 0.001). The time course of locomotor responses (Figure 3B) compares day 1 and day 5 after saline or cocaine injection. The Two-way repeated measures ANOVA showed differences in treatment (F3,100 = 28.63; *p* < 0.0001) and time (F5,100 = 28.63; *p* < 0.0001). Interactions between time and treatment were significantly different (F15,100 = 4.71, *p* < 0.0001). Bonferroni post-hoc showed significant differences between saline and cocaine treatment on day 1 until 40 min after injection (*p* < 0.01). Also, Bonferroni post-hoc showed significant differences between day 1 and 5 of cocaine injection after 40 min of the treatment (*p* < 0.001). Together, these results indicate that animals were successfully sensitized to cocaine.

In order to determine PKC’s role in Glu presynaptic terminals, sEPSCs were recorded from VTA DA neurons in brain slices of saline- and cocaine-treated animals preincubated for 30 min with phorbol 12-myristate 13-acetate (PMA; 0.5 µM; PKC activator). In addition, intracellular GÖ6976 application (PKC antagonist; 1 µM) via the pipette was used to exclude PKC postsynaptic actions. The brain slices of both treatment groups (saline and cocaine) were incubated in artificial cerebrospinal fluid (ACSF) without PMA as control. PMA increased sEPSCs frequency when compared to saline brain slices without PMA pretreatment (Figure 3C); Figure 3E,G show that PMA pretreatment shifts to the left the cumulative probability in the inter-event interval without changing the amplitude distribution, respectively. In contrast, sEPSCs sample recordings of PMA pretreated slices from cocaine-injected animals showed no differences in frequency or amplitude (Figure 3D,F,H). PMA pretreatment significantly increased the sEPSC frequency of VTA DA neurons from saline-treated animals (control 3.253 ± 0.9110 Hz, *n* = 12; PMA 13.05 ± 3.498 Hz, *n* = 13; unpaired *t*-test, *p* < 0.05; Figure 3I) but not the amplitude (control 14.54 ± 0.37 pA, *n* = 12; PMA 14.51 ± 0.49, *n* = 13; unpaired *t*-test, *p* = 0.95; Figure 3J). In slices from cocaine-injected animals, PMA pretreatment failed to increase the frequency (control 4.06 ± 0.88, *n* = 8; PMA 8.22 ± 3.16, *n* = 9; unpaired *t*-test, *p* = 0.24; Figure 3I) or amplitude (control 15.01 ± 0.43, *n* = 8; PMA 14.11 ± 0.40, *n* = 9; unpaired *t*-test, *p* = 0.15; Figure 3J). Altogether, these results demonstrate that presynaptic PKC activation may play a role in glutamate release decline after repeated cocaine injections.

### 2.4. Activation of α1-ARs Failed to Increase AMPA EPSCs Amplitude after a 7-Day Withdrawal Period

**Figure 4:**
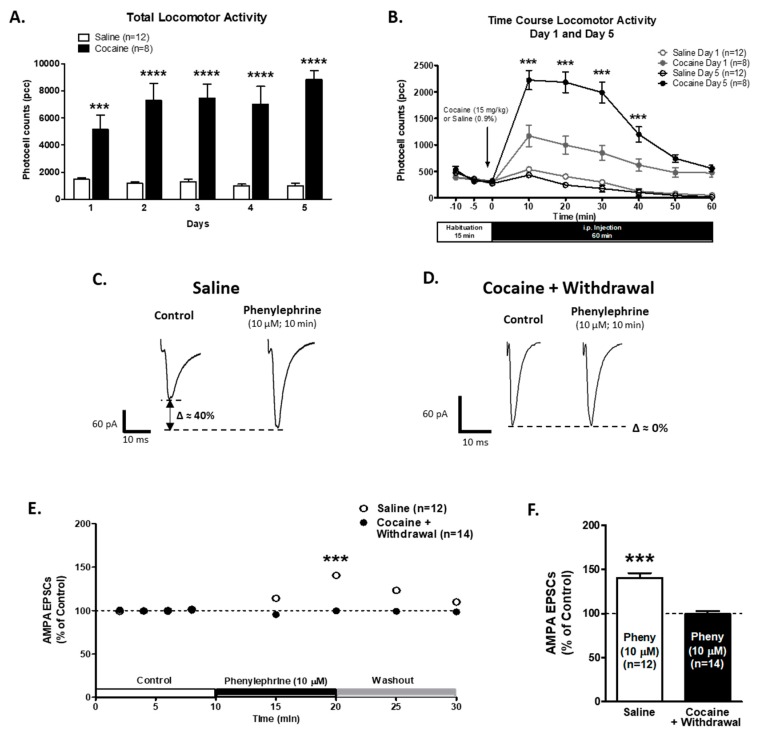
α1-ARs-mediated effect on AMPA EPSCs is still absent in VTA DA neurons after a 7-day withdrawal period. (**A**) A bar graph showing the total locomotor activity recorded from saline- (0.9%, white bars; *n* = 12) and cocaine- (15 mg/kg i.p., black bars; *n* = 8) treated animals. Cocaine-injected animals showed a progressive increase in locomotor activity over 5 days when compared to saline-injected animals (Two-way repeated measures ANOVA, day factor F5,72 = 3.28; *p* < 0.05; treatment factor F1,72 = 75.42, *p* < 0.0001). On day 5, the total activity was significantly increased when compared to day 1 in cocaine-treated animals (Bonferroni post hoc test *p* < 0.0001). (**B**) The time course of locomotor activity on days 1 (grey) and 5 (black) of cocaine- (15 mg/kg, i.p.; *n* = 8) (filled) or saline- (unfilled; *n* = 12) treated animals. Cocaine sensitization was manifested as an increase in locomotor activity during the first 40 min after cocaine injection in comparison with day 1. The data (pcc/60 min; Mean ± SEM) were analyzed by Two-way repeated measures ANOVA (treatment factor F3,180 = 72.73; *p* < 0.0001; time factor F5,180 = 110.2; *p* < 0.0001), Bonferroni post hoc test (*p* < 0.001). (**C**,**D**) Representative recordings from saline- and cocaine-treated animals after 7 days of withdrawal, respectively, illustrating that phenylephrine superfusion (10 μM, 10 min) induces a significant increment in AMPA EPSCs amplitude only in VTA DA neurons from saline-treated animals. (**E**) The time course summary of the effects of phenylephrine bath application on AMPA EPSCs amplitude recorded from 12 VTA DA neurons from saline-treated animals and 14 VTA DA neurons from cocaine-treated animals at 8 min of control (2 min intervals), 5 and 10 min phenylephrine (10 μM), and 5 and 10 min washout. A 10 min phenylephrine application increases the AMPA EPSCs amplitude only in neurons from saline-treated animals (Two-way repeated measures ANOVA, time factor F7,168 = 11.68, *p* < 0.0001; treatment factor F1,168 = 21.20, *p* < 0.001). A Bonferroni post hoc test demonstrated significant differences between saline and cocaine EPSCs recordings after 5 and 10 min of phenylephrine superfusion (*p* < 0.001). (**F**) A bar graph showing that, in neurons from saline-treated animals (*n* = 12/12), phenylephrine application resulted in a ~40% increase in AMPA EPSCs amplitude. No significant differences were observed on VTA DA neurons from cocaine-treated animals after 10 min of phenylephrine application when compared to control EPSCs recordings (99.6 ± 3.3%, *n* = 14/8, One-way ANOVA, Newman-Keuls post-hoc, *p* < 0.001). *** *p* < 0.001, **** *p* < 0.0001. *n* = the number of cells recorded/number of animals.

To evaluate if the cocaine induced alteration in α1-AR’s effect on AMPA EPSCs was still present after a withdrawal period, we recorded AMPA EPSCs on VTA DA neurons of saline- and cocaine-injected animals that went through a 7-day withdrawal period. Male rats were injected with either saline (0.9%; *n* = 12) or cocaine (15 mg/kg, i.p.; *n* = 8) for five consecutive days to induce behavioral sensitization [54]. Two-way repeated measures ANOVA showed significant differences in day factor (F5,72 = 3.28; *p* < 0.05) and treatment factor (F1,72 = 75.42, *p* < 0.0001) on the total locomotor activity of cocaine-treated animals in comparison to saline-treated animals (Figure 4A). The interaction between treatment and day showed significant differences (F4,72 = 5.30, *p* < 0.001). The Bonferroni post hoc test presented differences on all treatment days between saline and cocaine injected animals (Figure 4A).

The time course of locomotor responses (Figure 4B) compares day 1 and day 5 after saline or cocaine injection. Two-way repeated measures ANOVA showed differences in treatment (F3,180 = 72.73; *p* < 0.0001) and time (F5,180 = 110.2; *p* < 0.0001). The interaction between treatment and time showed differences (F15,180 = 17.54; *p* < 0.0001). The Bonferroni post hoc analysis demonstrated an increase in locomotor activity after 5 days of cocaine treatment during the first 40 min after cocaine injection compared to their first cocaine treatment (*p* < 0.0001). These results indicate that the animals used in this study were successfully sensitized to cocaine.

After a 7-day withdrawal period, electrophysiological recordings of the VTA DA neurons of saline-injected animals treated with the α1-AR agonist (phenylephrine, 10 μM; 10 min) showed an increase in AMPA EPSCs peak amplitude to 140.5 ± 5.4% of control (*n* = 12; One-way ANOVA F2,38 = 26.57, *p* < 0.001, Figure 4C,E,F). However, in the slices from animals treated with cocaine for 5 days, which were submitted to a 7-day withdrawal period, phenylephrine superfusion was unable to exert its excitatory action on AMPA EPSCs after 5 or 10 min of application (control 100.2 ± 1.6%; phenylephrine 10 min 99.6 ± 3.3%; *n* = 14; One-way ANOVA F2,42 = 0.97, *p* = 0.38, Figure 4 D–F). Two-way repeated measures ANOVA showed significant differences in time (F7,168 = 11.68, *p* < 0.001 and treatment factor (F1,168 = 21.20, *p* < 0.001). The interaction between time and treatment showed significant differences (F7,168 = 12.42, *p* < 0.0001). The Bonferroni post hoc test demonstrated significant differences between saline and cocaine EPSCs recordings on 5 and 10 min of phenylephrine superfusion (*p* < 0.0001). These results strongly suggest that α1-ARs are still desensitized after 7 days of cocaine withdrawal.

### 2.5. α1-ARs Activation Decrease GABA_A_ IPSCs Amplitude in VTA DA Neurons from Acute Saline and Cocaine-Treated Animals

**Figure 5:**
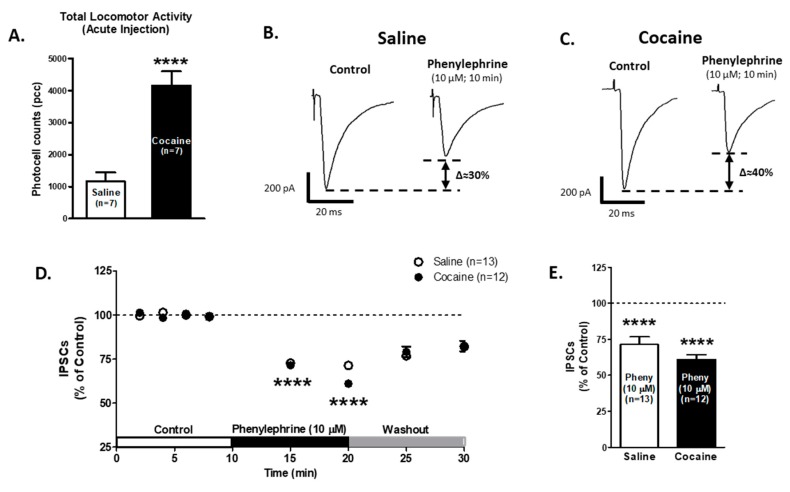
α1-ARs-mediated effect on GABA_A_ IPSCs amplitude is not affected in VTA DA neurons after one saline or cocaine injection. (**A**) A bar graph showing the total locomotor activity recorded from saline- (0.9%, white bar; *n* = 7) and cocaine- (15 mg/kg i.p., black bar; *n* = 7) treated animals. The locomotion of cocaine treated animals was significantly increased compared to saline-treated controls (Unpaired *t*-test, *p* < 0.0001). (**B**,**C**) Representative recordings from saline- and cocaine-treated animals, respectively, show that phenylephrine superfusion (10 μM, 10 min) induces a significant decrease in GABA_A_ IPSCs amplitude in VTA DA neurons from both groups. (**D**) The time course summary of phenylephrine effects on GABA IPSCs recorded from VTA DA neurons from saline- (*n* = 13) and cocaine- (*n* = 12) treated animals at 8 min of control (2 min intervals), 5 and 10 min phenylephrine (10 μM), and 5 and 10 min washout. A 10 min phenylephrine application decreases the GABA_A_ IPSCs amplitude in neurons from saline- and cocaine-treated animals (One-way ANOVA, Newman-Keuls post hoc test, *p* < 0.0001). (**E**) A bar graph showing that phenylephrine application results in a ~30% decrease in GABA_A_ IPSCs amplitude in VTA DA neurons from saline- (*n* = 13/7) and cocaine- (*n* = 12/7) treated animals (One-way ANOVA, Newman-Keuls post-hoc, *p* < 0.0001). **** *p* < 0.0001. *n* = the number of cells recorded/number of animals.

To study the presynaptic α1-AR modulation of GABA release onto VTA DA neurons after an acute cocaine injection, male rats were injected with either saline (0.9%; *n* = 7) or cocaine (15 mg/kg, i.p.; *n* = 7) for one day. The total activity (Figure 5A) showed differences between saline- (*n* = 7; 1166 ± 280.7 photocell counts) vs. cocaine- (*n* = 7; 4158 ± 439.5 photocell counts, Unpaired *t*-test, df = 12, t = 5.73, *p* < 0.0001) treated animals. These results indicate that acute cocaine-treated animals presented the characteristic increased locomotion response to the drug.

Phenylephrine application (10 μM) on VTA DA neurons decreased GABA_A_ IPSCs amplitude after 10 min, saline group: 71.3 ± 5.2% of control (*n* = 13; One-way ANOVA F2,36 = 17.75, Newman-Keuls post hoc test *p* < 0.0001, Figure 5B,D,E); cocaine group: 61.0 ± 3.1% of control (*n* = 12; One-way ANOVA F2,34 = 32.74, Newman-Keuls post hoc test *p* < 0.0001, Figure 5C–E). The two-way repeated measures ANOVA showed significant differences in time factor (F7,161 = 30.23, *p* < 0.0001) and no significant differences in treatment factor (F1,16 = 0.49, *p* = 0.48). The interaction between the treatment and time showed no differences (F7,161 = 0.53, *p* = 0.80). The Bonferroni post hoc test did not present differences between saline and cocaine IPSCs recordings (*p* > 0.05). These results suggest that α1-ARs modulation on VTA GABA neurotransmission is functional after one cocaine injection.

### 2.6. Modulation of α1-ARs on GABA Release is Present in the VTA DA Neurons of Sensitized Animals

**Figure 6:**
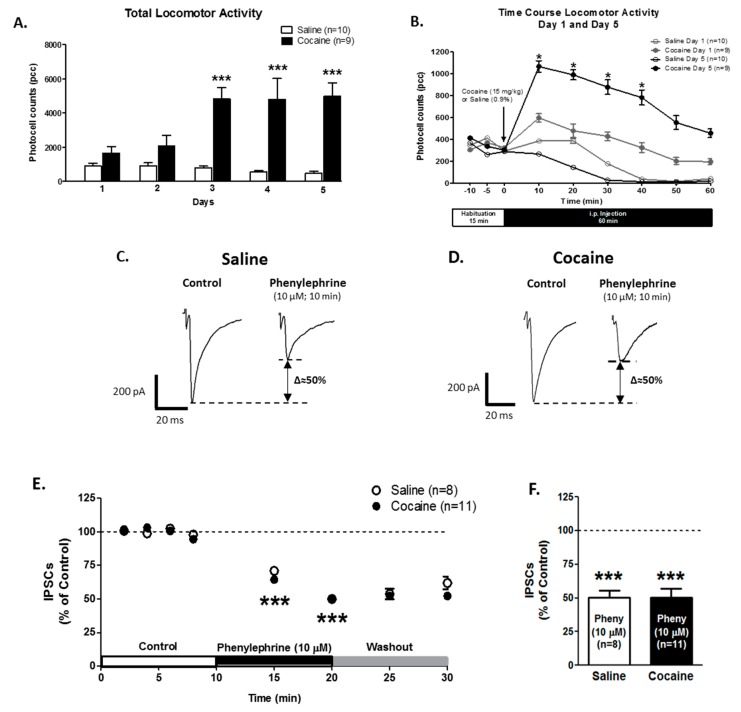
α1-ARs-mediated GABA_A_ IPSCs effect is present in VTA DA neurons from cocaine-sensitized animals. (**A**) A bar graph showing total locomotor activity recorded from saline- (0.9%, white bars; *n* = 10) and cocaine- (15 mg/kg i.p., black bars; *n* = 9) treated animals. Cocaine-injected animals showed a progressive increase in locomotor activity over 5 days. On days 3, 4, and 5, the total activity was significantly increased when compared to day 1 in cocaine-treated animals (Two-way repeated measures ANOVA, Bonferroni post hoc test, *p* < 0.001). (**B**) The time course of locomotor activity on days 1 (grey) and 5 (black) of cocaine- (15 mg/kg, i.p.) (filled) or saline- (unfilled) treated animals. Cocaine sensitization was manifested as an increase in locomotor activity during the first 10–40 min after cocaine injection in comparison with day 1. The data (pcc/60 min; Mean ± SEM) were analyzed by Two-way repeated measures ANOVA, Bonferroni post hoc test (*p* < 0.05). (**C**,**D**) Representative recordings from saline- and cocaine-treated animals, respectively, illustrate that phenylephrine superfusion (10 μM, 10 min) induces a significant reduction on GABA_A_ IPSCs amplitude in VTA DA neurons from saline- and cocaine-treated animals. (**E**) The time course summary of the effects of phenylephrine bath application on GABA_A_ IPSCs amplitude, recorded from 8 VTA DA neurons from saline-treated animals and 11 VTA DA neurons from cocaine-treated animals at 8 min of control (2 min intervals), 5 and 10 min phenylephrine (10 μM), and 5 and 10 min washout. A 10 min phenylephrine application increases the GABA_A_ IPSCs amplitude in neurons from saline- and cocaine-treated animals (One-way ANOVA, Newman-Keuls post hoc test, *p* < 0.001). (**F**) A Bar graph showing that, in neurons from saline- (*n* = 8/10) and cocaine- (*n* = 11/9) treated animals, phenylephrine application resulted in a ~50% reduction on GABA_A_ IPSCs amplitude (One-way ANOVA, Newman-Keuls post-hoc, *p* < 0.001). * *p* < 0.05; *** *p* < 0.0001. *n* = the number of cells recorded/number of animals.

For total activity (Figure 6A), Two-way repeated measures ANOVA showed differences in treatment: saline (*n* = 10) vs. cocaine (*n* = 10) (F1,58 = 35.01; *p* < 0.0001). Additionally, the total activity between the days showed differences (F4,68 = 5.33; *p* = 0.0009). The interaction between treatment and days showed differences (F4,68 = 8.01; *p* <0.0001). The Bonferroni post hoc analysis revealed that cocaine-treated animals had a higher total activity than saline-treated animals on days 3 (*p* = 0.0001), 4, and 5 (*p* = 0.0001). The time course of locomotor responses (Figure 6B) compares day 1 and day 5 after saline or cocaine injection. The Two-way repeated measures ANOVA showed differences in treatment (F3,150 = 13.21; *p* < 0.0001) and time (F5,150 = 39.44; *p* < 0.0001). The interactions between time and treatment showed significant differences (F15,150 = 2.75, *p* = 0.0009). The Bonferroni post hoc analysis demonstrated an increase in locomotor activity after 5 days of cocaine treatment for 40 min after cocaine injection compared to their first cocaine treatment (*p* < 0.05). Together, these results indicate that the animals used in this study were successfully sensitized to cocaine.

In slices from saline-treated animals, bath application of the selective α1-AR agonist, phenylephrine (10 μM) after 10 min, decreased GABA_A_ IPSCs amplitude to 50.0 ± 5.4% of control (*n* = 8; One-way ANOVA F2,24 = 62.61, Newman-Keuls post hoc test, *p* < 0.0001, Figure 6C,E,F). Similarly, 10 min of phenylephrine superfusion decreased GABA_A_ IPSCs amplitude to 50.3 ± 6.9% of control (*n* = 11; One-way ANOVA F2,30 = 27.05, Newman-Keuls post hoc test, *p* < 0.0001, Figure 6D–F) in slices from animals treated with cocaine for 5 days. No significant differences were found by Two-way repeated measures ANOVA in the treatment factor (F1,119 = 0.32, *p* = 0.57), nonetheless, the time factor was significantly different (F7,119 = 56.12, *p* < 0.0001). The interaction between time and treatment showed no significant differences (F7,119 = 0.48, *p* = 0.84). Additionally, the Bonferroni post hoc test showed no differences between saline and cocaine IPSCs recordings (*p* > 0.05). These results demonstrate that α1-ARs modulation on GABA neurotransmission is present after 5 days of cocaine administration.

## 3. Discussion

The cocaine sensitization paradigm modifies the α1-ARs activation of glutamatergic, but not GABAergic, transmission. The present results showed that α1-AR-mediated activation on glutamatergic transmission is no longer present after 5 days of cocaine sensitization protocol, even after withdrawal period. In addition, presynaptic the PKC activity related to glutamatergic transmission decreased in VTA neurons of cocaine-sensitized animals. On the other hand, α1-AR- regulation of GABAergic transmission is unaffected by acute and repeated cocaine treatment. As a possible limitation of this study future investigations should address sex differences in the observed responses.

Cocaine rapidly increases monoamines (dopamine, serotonin, and norepinephrine) concentration in the synaptic cleft by blocking their reuptake [55,56]. A single injection or repeated cocaine administration increases glutamatergic and decreases GABAergic synaptic transmission onto VTA DA neurons [40,57]. Changes on glutamatergic and GABAergic neurotransmission after cocaine injection could be due, in part, to norepinephrine action on α1-ARs. Therefore, α1-ARs activation, after cocaine injection, could modify VTA DA neuronal excitability and increase DA release onto the nucleus accumbens (NAcc) [45]. In accordance with this, the present results show that α1-ARs activation increases glutamatergic transmission onto VTA DA neurons 24 h after an acute cocaine injection. This activation of α1-ARs could generate positive feedback onto VTA DA cells that might be important for the effects induced by drug abuse [35,40,45,58]. We have shown that α1-ARs increase presynaptic glutamate release onto VTA DA neurons [12]. Thus, after an acute cocaine injection, presynaptic α1-ARs activation could help promote long-term potentiation (LTP) at VTA DA neurons synapses via increases in extracellular glutamate. Cocaine-induced synaptic plasticity in the VTA has been associated with maladaptive behaviors and the development of addiction [59,60,61,62]. Long-term changes in the VTA are induced by a single and multiple cocaine injections [40,41]. In addition, LTP, as the persistent increase in AMPA/NMDA ratio on VTA DA cells, is critical for the maintenance of cocaine sensitization [63]. These changes in synaptic plasticity in the VTA have been implicated in the development of drug addiction and cocaine sensitization [5,54,64,65].

Different studies have shown that α1-ARs have a critical role in drug addiction. Pre-exposure to prazosin (α1-ARs antagonist) decreases behavioral sensitization and psychostimulants’ self-administration [19,49,66]. Similar results were obtained with α1b-AR knockout animals [47,48]. An alteration in α1-ARs activation inhibits DA release on NAcc after D-amphetamine systemic administration [66,67]. These findings indicate that α1-ARs activation is an important substrate in the development of drug addiction to psychostimulants. Our results show that α1-ARs activation failed to increase glutamatergic transmission onto VTA DA neurons in sensitized animals. Nalepa et al., [52] indicated that cocaine sensitization was associated with changes in α1-ARs density in certain regions of rats’ brains. Although this study does not show results on α1-ARs at the VTA, it suggests that these receptors are susceptible to changes and therefore their effect could be absent after cocaine sensitization. We postulate that after the initiation of cocaine sensitization, α1-ARs located on glutamatergic afferents to VTA DA neurons are desensitized. Receptor desensitization is described as a decrease in the response to an agonist after persistent stimulation and constitutes a regulation process of many G-protein coupled receptors [68,69,70,71,72]. The desensitization process involves the stimulation of G-protein coupled receptor kinases, β-arrestins, and PKC activation that induces inactivation and receptor internalization [68,70]. α1-ARs desensitization has been extensively described [69,71,73,74]. This process could be a homeostatic response to prevent prolonged receptor stimulation and the detrimental effects associated with this protracted stimulation.

The blockade of α1-ARs or 5-HT_2A_ receptors only attenuates the behavioral response to cocaine, D-amphetamine, and morphine administration [19,45,47,48]. In addition, the simultaneous blockade of α1-ARs and 5-HT2A receptors entirely abolish the locomotor response and development of behavioral sensitization to psychostimulants and opiates [48]. Therefore, α1-ARs and 5-HT_2A_ receptors appear to be physiologically interrelated and could mediate the locomotor response and development of behavioral sensitization to psychostimulants and opiates. On the other hand, studies have shown that repeated cocaine injections produce a persistent increase in the capacity of cocaine to elevate extracellular glutamate levels via an increase in D1 receptor stimulation of glutamate release at the VTA [75]. The α1-ARs desensitization after repeated cocaine administration could induce a shift towards neural pathways controlled by other receptors. Thus, the increase in glutamatergic transmission that was mediated by presynaptic α1-ARs could be compensated by 5-HT_2A_ or D1 receptors’ activation.

Here, we demonstrate that presynaptic PKC activation, in the presence of phorbol ester (PMA), did not increase glutamate release onto VTA DA neurons after chronic cocaine exposure. In agreement with our results, it has been shown that PKC increases its activity after an acute cocaine injection but not after chronic (7 days) cocaine administration [76,77]. Studies have also demonstrated that cocaine temporarily increases PKC activity in the VTA. Repeated cocaine exposure increases PKC activity at 2 but not 6 or 24 h after the last injection [76,77]. No significant cocaine-induced changes in PKC activity in the VTA were detected 24 h after a cocaine challenge injection [76]. In VTA DA neurons from naïve animals, PKC activation with a phorbol ester causes an increase in both mEPSC frequency and amplitude [78]. Altogether, these data suggest that repeated cocaine injection induces a transitory increase (2 h but not 6 or 24 h) in PKC activity in the VTA during the initiation of cocaine sensitization. Also, the modification of PKC activity could be part of the transient processes that occur at the VTA during the initiation of cocaine sensitization. Therefore, our results suggest that changes in PKC activity in the VTA after repeated cocaine injections may be involved in the initiation of behavioral sensitization.

The desensitization of α1-AR has been shown to be associated with decreases in the number of receptor sites due to prolonged exposure to NE or agonists (phenylephrine), which results in decreased responsiveness [74]. Our results show that after 7 days of withdrawal, α1-AR-mediated increased glutamatergic transmission onto VTA DA neurons is still absent. Therefore, the increased NE in the synaptic cleft induced by cocaine administration could overstimulate and cause a long-lasting desensitization of α1-ARs.

The desensitization of α1-AR showed here could be part of several neuroadaptations induced at the mesocorticolimbic system by chronic cocaine treatment. Some of these neuroadaptations include: (A) an increase in GluR1 subunit with increases in AMPA/NMDA ratio on VTA DA cells (5–7 days after cessation of drug administration) [41,43,79,80]; (B) an increased glutamate transmission [75]; (C) a reduced D1-stimulated GABA transmission in the VTA [81]; (D) enhanced basal levels of extracellular DA [54]; (E) increased tyrosine hydroxylase synthesis [82]; and (F) a decrease in I_h_ current and DA neurons capacitance [83,84]. In addition, a decrease in nerve terminal glutamate immunoreactivity within the VTA in cocaine sensitization, after a withdrawal period, has been reported [85,86,87]. Most of these modifications are temporary but necessary to trigger other alterations for the maintenance and expression of addiction-related behaviors [34,88,89].

In contrast to our results of α1-AR modulation on glutamatergic neurotransmission after cocaine treatment, a decrease in GABAergic transmission onto VTA DA neurons by α1-AR activation is still observed following both the acute injection and 24 h after the initiation of cocaine sensitization. Cocaine administration decreases evoked GABA_A_ currents on VTA neurons [43,90]. Other investigators have shown that different addictive drugs, including cocaine, attenuated long-term potentiation of GABAergic synapses onto VTA dopamine neurons [91]. In addition, it has been demonstrated that increased GABA neurotransmission attenuates cocaine behavioral sensitization [92]. Therefore, after drug administration, DA neurons could be subjected to less inhibition and consequently have increased excitability in response to a given stimulus.

Our results on naïve rats demonstrate that α1-ARs modulation on GABAergic release is dependent on voltage-sensitive sodium channels, since there are no changes in the frequency or amplitude of mIPSCs (in the presence of TTX) [13]. Steffesen et al. [93], demonstrated that cocaine reduced GABA release onto VTA DA neurons by blocking voltage-sensitive sodium channels present on presynaptic GABAergic terminals. Thus, cocaine-induced NE increase can activate α1-ARs and diminish GABA release onto VTA DA neurons.

The stimulation of GABA neurons at the rostromedial tegmentum (RMTg) increases IPSCs’ amplitude in VTA DA cells that project to the NAcc lateral shell [94]. The VTA DA–NAcc lateral shell connection is highly associated with reward behavior [3,95]. The activation of VTA GABA neurons in VTA DA cells has been linked with the disruption of reward behaviors [96]. Taken together, these data suggest that the activation of GABA-RMTg inputs and local VTA GABA neurons can induce an inhibition on VTA DA cells and disturb reward-related behaviors. Consequently, a decrease in these two inhibitory sources onto VTA DA cells can facilitate the development of reward behaviors. After acute and repeated cocaine injections, α1-ARs activation induces a decrease in GABA neurotransmission onto VTA DA neurons. Therefore, we can hypothesize that the decrease in GABA release via α1-AR activation can reverse neurotransmitter actions related to aversive behaviors, allowing neurons to increase their excitation. This increased excitability will enhance DA release in the NAcc and facilitate the development of rewarding behaviors.

**Figure 7:**
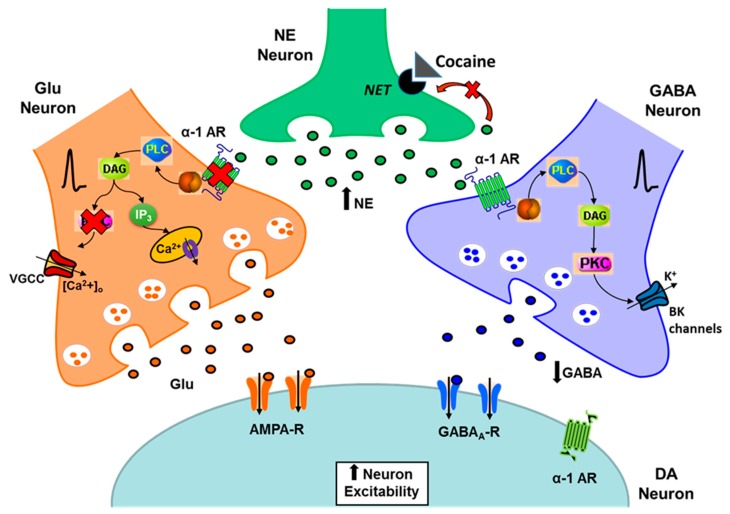
Proposed model for α1-ARs action on glutamatergic and GABAergic terminals during the initiation and expression of cocaine sensitization. After cocaine sensitization and a withdrawal period, the α1-ARs present on glutamate terminals on VTA DA neurons are desensitized. In addition, a PKC-mediated increase in glutamate release on VTA DA cells is absent after 5 days of cocaine injections. In contrast, the α1-AR-mediated decrease on GABA release on VTA DA neurons remains unaltered.

In conclusion, the present data show that, after the initiation of cocaine sensitization, the α1-ARs present on glutamatergic terminals onto VTA DA neurons are desensitized and their PKC-mediated response is absent. Additionally, this receptor desensitization is still present after a withdrawal period. On the other hand, the α1-ARs modulation of GABA neurotransmission remains intact after chronic cocaine treatment (Figure 7). Therefore, the cocaine-induced enhanced DA cell excitability could be increased through a reduction of GABA inhibition on VTA DA neurons even if the α1-ARs function on glutamate release and PKC activation is not present. Altogether, these changes in α1-ARs neuromodulation could be part of potential homeostatic alterations occurring at the VTA after the initiation of cocaine sensitization. The understanding of the neuropathological mechanisms and the neurochemical imbalances that are behind cocaine addiction could help in the development of effective pharmacological treatments.

## 4. Materials and Methods 

### 4.1. Animals

All experimental procedures were performed according to the US Public Health Service publication “Guide for the Care and Use of Laboratory Animals” and were approved by the Animal Care and Use Committee at the University of Puerto Rico Medical Sciences Campus (IACUC# 4050108; 9/4/2014)). Sprague-Dawley male rats weighing between 250–300 g were housed two per cage and maintained at a constant temperature and humidity with a 12-h hour light/dark cycle. Water and food were provided ad libitum throughout the course of the experiment.

### 4.2. Sensitization Protocol and Behavior

The animals were divided randomly into two groups (saline and cocaine). Two days before the beginning of the experiment, each group was habituated (for 1 h) to the infrared photocell box. On experimental day one, the animals were placed for 15 min in the photocell box. After the 15-min habituation, the animals were treated with either 15 mg/kg i.p. of cocaine (Sigma, St. Louis, MO, USA) or isovolumetric saline injections. Immediately after the injections, locomotor activity was recorded at 10 min intervals for 60 min. This procedure was repeated for five (5) consecutive days. A significant statistical difference of *p* < 0.05 between day 1 and day 5, (Two-way ANOVA) was considered a successful sensitization protocol [33]. Twenty-four hours after the last cocaine injection or following the 7-day withdrawal period, animals were anesthetized with a 90 mg/kg of chloral-hydrate i.p. (Sigma, St. Louis, MO, USA) and their brains rapidly removed.

### 4.3. Electrophysiological Studies

EPSCs and IPSCs from the VTA DA neurons of rats previously treated with 1 or 5 injections of cocaine or saline were measured using whole cell voltage clamp techniques. Phenylephrine superfusion was used to determine whether the cocaine-induced potentiation of AMPA or GABA_A_ mediated currents are altered by α1-AR [12,13,16]. To determine the effect of cocaine sensitization on presynaptic PKC activation, slices were preincubated for 30 min with phorbol 12-myristate 13-acetate (PMA; 0.5 µM). Also, an internal solution containing GO6976 (PKC antagonist; 1 µM) via the recording pipette was used to exclude PKC postsynaptic actions.

### 4.4. Slice Preparation

Horizontal slices (220 µM) containing the VTA were cut using a vibratome (VT1000S, Leica, Wetzlar, Germany). The rat midbrain was placed on an ice-cold oxygenated artificial cerebrospinal fluid (ACSF) containing (in mM) 127 NaCl, 2.5 KCl, 1.25 NaH_2_PO_4_, 25 NaHCO_3_, 2 CaCl_2_, 1 MgCl_2_, and 25 D^(+)^-glucose and was saturated with a 95%O_2_–5%CO_2_ gas mixture to a pH = 7.4. Slices were transferred to an intermediate chamber and incubated at 32 °C in the same solution for 45 min before the initiation of electrophysiological recordings. MK-801 (10 μM, Tocris, Ellisville, MO, USA) was added to the incubation solutions to block N-methyl-D-aspartate (NMDA)-mediated excitotoxicity.

### 4.5. Electrophysiological Recordings

VTA slices were totally submerged in a recording chamber (500 µL) with ACSF superfusion at 1–2 mL/min at 32°C. Whole cell voltage clamp recordings were obtained from visually identified neurons in the VTA using an infrared microscope with differential interference contrast (DIC) optics (BX51WI Olympus, Japan). The recordings were acquired through data acquisition software (pClamp 10, Molecular Devices, Sunnyvale, CA, USA). All recordings were performed in putative DA neurons identified by the presence of a large hyperpolarization-activated cation current (I_h_ > 200 pA), evoked by 1-s hyperpolarizing steps from −60 to −130 mV. I_h_ is present in about 84% VTA DA neurons and VTA GABA cells rarely express this conductance [97,98]. Therefore, the contribution of non-dopaminergic neurons to the experimental recordings performed in this study is not likely to be significant. Whole-cell voltage-clamp recordings were made at a holding potential of −70 mV unless otherwise indicated.

*AMPA Excitatory Postsynaptic Currents (EPSCs) Recordings*: Picrotoxin (100 μM, Sigma, St. Louis, MO, USA) was added to the ACSF during the recording procedures to block GABA_A_ receptor-mediated inhibitory postsynaptic currents (IPSCs). Borosilicate glass patch pipettes (O.D.1.5 mm, I.D:1,0 mm WPI, Sarasota, FL, USA) were pulled to a final resistance of 3–6 MΩ and filled with (in mM): 115 CH3SO4K (Methyl potassium sulfate); 20 KCl; 1.5 MgCl2; 5 HEPES; 1 EGTA; 2 ATP; 0.2 GTP; 10 Creatine Phosphate (CP); pH 7.25, 290 mOsm. (Na) GTP, (Mg) ATP, and (Na) CP were added fresh daily.

*GABA_A_ Inhibitory Postsynaptic Currents (IPSCs) Recordings*: The superfusion medium contained 2-amino-5-phosphonopentanoic acid (AP5; 100 μM) and either 6-cyano-2,3-dihydroxy-7 nitroquinox saline (CNQX; 10 μM) or 6,7-Dinitroquinoxaline-2,3-dione (DNQX; 10 μM) to block fast NMDA- or AMPA-mediated synaptic potentials, respectively. In all experiments, eticlopride (100 μM) was included in the superfusion solution to block any possible effects mediated by the dopamine D2 receptor. Whole-cell voltage-clamp recordings were made using microelectrodes filled with a solution containing (in mM): 70 K+ gluconate, 80 KCl, 1 EGTA, 5 HEPES, 2 MgATP, and 0.3 GTP. At the end of each experiment, picrotoxin (100 μM) was added to completely abolish all evoked or spontaneous GABA_A_ mediated postsynaptic currents.

The data were collected through a Multiclamp 700B amplifier (Axon Instruments, Foster City, CA, USA), filtered at 1 kHz, digitized at 5 kHz using Digidata 1440A (Axon Instruments, Foster City, CA, USA), and stored in a PC computer and analyzed off-line using GraphPad Prism 5 (GraphPad Software, Inc) software. The pipette’s liquid junction potential was offset and compensated using standard Multiclamp 700B circuitry. The seal’s quality used was 4–6 GΩ. The series resistances were not compensated and were monitored during the entire experiment. The data were discarded if changes (in seal) of more than 15% occurred.

### 4.6. Recording of Synaptic Currents

A bipolar stainless-steel stimulating electrode (FHC Inc, Bowdoin, ME, USA) was placed approximately 100 μm rostral to the recording electrode and was used to stimulate afferents at 0.1Hz by applying a brief (400 μs; low pass filter 1 kHz, digitized 5 KHz) electrical pulse (100–300 μA). AMPA EPSCs and GABA_A_ IPSCs were recorded at −70 mV. All EPSCs and IPSCs shown in the figures are averages of 5 current traces. AMPA EPSCs’ and GABA_A_ IPSCs’ amplitudes were calculated by taking a 1 ms window around the peak of the EPSC or IPSC and comparing this to a 5 ms window immediately before the stimulation artifact. Peak EPSCs’ or IPSCs’ amplitudes were averaged during the control recordings. This value was used to normalize control and treatment recordings. This procedure allowed data recording as percentages of the control condition for appropriate statistical comparisons. Spontaneous AMPA EPSC’s (sEPSCs) signals were recorded at −70 mV, filtered at 1 kHz and digitized at 5 kHz using pCLAMP 10 software (Molecular Devices, Sunnyvale, CA, USA). For a given cell, sEPSCs were collected (1 sweep for each condition, 3min/sweep) for a control and phenylephrine’s period. The recorded sEPSCs were analyzed afterward using Mini Analysis program 6.0.7 (Synaptosoft Inc. Decatur, GA, USA). Detection criteria were set at >6 pA, <1.3 ms rise time, and <0.1 ms decay time. The choice of this cutoff amplitude for the acceptance of sEPSCs was made to obtain a high signal-to-noise ratio. Each event was also visually inspected to prevent noise disturbance during the analysis.

### 4.7. Drugs

Cocaine hydrochloride, phenylephrine hydrochloride ([R]-[−]-1-[3-Hydroxyphenyl]-2-methylaminoethanol hydrochloride), phorbol 12-myristate 13-acetate (PMA) were purchased from Sigma–Aldrich (St. Louis, MO, USA), GO6976 was purchased from Tocris (Ellisville, MO, USA).

### 4.8. Data Analysis

All data were presented as mean ± SEM. The statistical significance was assessed using an Unpaired *t*-test, One-Way ANOVA with Newman-Keuls as a post hoc analysis or Two-Way repeated measures ANOVA with time and treatment as factors, and Bonferroni as a post hoc analysis, except for the significance of horizontal shifts to the cumulative probability distribution plots obtained from single cell recordings. For the latter case, the Kolmogorov–Smirnov test was used. *p* values were reported throughout the text and the significance was set at *p* < 0.05.

## Abbreviations

α1-AR	Alpha 1 Adrenergic Receptor
µM	Micromolar
5-HT	5-hydroxytryptamine
ACSF	Artificial cerebrospinal fluid
AMPA	2-Amino-3-(5-Methyl-3-Oxo-1, 2-Oxazol-4-yl) Propanoic Acid
DA	Dopamine
EPSC	Inhibitory postsynaptic current
GABA	Gamma-aminobutyric acid
Glu	Glutamatergic
i.p.	Intraperitoneal
I_h_	H current
IPSC	Inhibitory postsynaptic current
kg	Kilogram
LTP	Long-term potentiation
mg	Milligram
min	Minutes
ms	Milliseconds
NAcc	Nucleus Accumbens
NE	Norepinephrine
NMDA	*N*-Methyl d-Aspartate
pcc	Photocell counts
PKC	Protein kinase C
PMA	Phorbol 12-myristate 13-acetate
RMTg	Rostromedial tegmentum
sEPSC	Spontaneous excitatory postsynaptic current
VTA	Ventral Tegmental Area
